# Why do children under 5 years go to the GP in Lambeth: a cross-sectional study

**DOI:** 10.1136/bmjopen-2023-082253

**Published:** 2024-05-23

**Authors:** Eleanor May Craven, Gemma Luck, David Whitney, Hiten Dodhia, Shaneka Foster, Carla Stanke, Paul T Seed, James Crompton, Kerry Ann Brown

**Affiliations:** 1 Liverpool University Hospitals NHS Foundation Trust, Liverpool, UK; 2 National Children's Bureau, London, UK; 3 Department of Population Health Sciences, School of Life Course & Population Sciences, King's College London, London, UK; 4 Public Health, London Borough of Lambeth, London, UK; 5 Department of Women & Children's Health, School of Life Course & Population Sciences, Kings College London, London, UK; 6 University of Exeter Medical School, University of Exeter, Exeter, UK; 7 Faculty of Epidemiology and Population Health, London School of Hygiene and Tropical Medicine, London, UK

**Keywords:** community child health, health equity, public health, paediatrics, primary health care

## Abstract

**Abstract:**

**Objectives:**

This study identifies the most common recorded reason for attendance to primary care for children under 5 years old, including a breakdown via age, ethnicity, deprivation quintile and sex.

**Design:**

Cross-sectional.

**Setting:**

39 of 40 general practices in Lambeth, London, UK.

**Participants:**

22 189 children under 5 years who had attended primary care between the 1 April 2017 and 31 March 2020 and had not opted out of anonymous data sharing within Lambeth DataNet.

**Outcome measure:**

The primary objective was to identify the most frequently recorded complaint in general practice for children under 5 years old. The secondary objective was to understand how presenting complaint differs by age, ethnicity, sex and deprivation level. The third objective was to create a multivariate logistic regression with frequent attendance as the outcome variable.

**Results:**

Nine conditions formed over 50% of all patient interactions: the most common reason was upper respiratory tract infections (14%), followed by eczema (8%) and cough (7%). While there was some variation by ethnicity and age, these nine conditions remained dominant. Children living in the most deprived area are more likely to be frequent attenders than children living in the least deprived area (adjusted OR (AOR) 1.27 (95% CI 1.14 to 1.41)). Children of Indian (AOR 1.47 (1.04 to 2.08)), Bangladeshi (AOR 2.70 (1.95 to 3.74)) and other white (AOR 1.18 (1.04 to 1.34)) ethnicities were more likely to be frequent attenders, compared with those of white British ethnicity.

**Conclusions:**

Most reasons for attendance for children under 5 years to primary care are for acute, self-limiting conditions. Some of these could potentially be managed by increasing access to community care services, such as pharmacies. By focusing on the influence of the broader determinants of health as to why particular groups are more likely to attend, health promotion efforts have the opportunity to reduce barriers to healthcare and improve outcomes.

**STRENGTHS AND LIMITATIONS OF THIS STUDY**

- The study location and large dataset enabled detailed analysis by ethnicity, which has been missing from the literature.
- Creation of a novel coding schema of general practitioner records enabled analysis close to the data that minimised inconsistencies in coding practices.
- The definition of frequent attendance did not include children who did not attend primary care within the time period, missed appointments or routine appointments, such as for vaccinations.
- There may be multiple codes in a single appointment relating to the same presenting complaint, resulting in overcounting; however, the impact of this appears to be minimal, as only a minor difference was seen in percentage frequency between analyses using a single code per appointment or using all codes (without duplicates).
- The most significant limitation of this study is the inability to distinguish differences between the burden of disease and differences in health services use.

## Introduction

 Primary care is considered central to the functioning of the health system in the UK and is the location of over 90% of National Health Service (NHS) health contacts.^1 2^ However, general practices are ‘reaching breaking point’, with data from NHS digital showing practices in England under pressure: delivering nearly 30 million appointments in March 2024, 1.5 million more than in March 2021 and 4.3 million more than in March 2019.^3 4^ This is due to a combination of the influence of the COVID-19 pandemic, possible underinvestment in community health services, growing demands from an increasing population and chronic staffing shortages.^3 5 6^ There is a need to consider how to maximise available resources and training to sustain quality care within primary care, in order to maintain and improve this service.

Children under 5 years have among the highest consultation rates of any age group in primary care in the UK, other than older people.^2^ Despite this, the majority of research into paediatric use of primary care focuses on the epidemiology of single diseases, rather than the overall burden of illness.^2 7^ This limits health promotion opportunities by perpetuating the so-called ‘silo-mentality’, which neglects to consider issues within context, reducing interorganisational communication and cooperation, and preventing a cohesive approach to tackle broader socioeconomic determinants of health.^8^

An Australian study found that only 30 conditions made over 50% of a general practitioner’s (GP) overall workload.^9^ Should a small number of paediatric conditions create a similarly large illness burden upon general practices in the UK, it could then form a target for health promotion interventions. If these services were better adapted to this age group, it could make them more efficient, reducing waiting times, and improve patient outcomes.^10^ In addition, 40–50% of GPs have no formal training in paediatrics in many parts of the country, culminating in many GPs lacking the specialist training and experience to treat children effectively.^11^ This is despite the fact that 25% of their patients are children and up to 40% of consultations are with children and families.^2 11^ This results in unnecessary referrals and unsustainable increasing demands on secondary care.^1 12^ Therefore, understanding primary care usage will enable better support for GPs, improve treatment within primary care and provide an opportunity for more efficient, stronger multidisciplinary teamwork.

Timely and equitable access to primary care is considered one of the most feasible ways to reduce health inequality; however, inequalities exist within the use of primary care itself.^13^ There is an association between greater deprivation and frequent attendance to primary care; despite this, or perhaps because of this, deprivation is also linked to poorer experiences within primary care, including lower patient satisfaction, shorter consultations, lower rates of specialist referral, less and weaker medication prescribed, and delayed diagnosis.^14^,^16^ There are also significant racial inequalities within healthcare access, the reasons for which are numerous and include: lack of familiarity with healthcare systems; experiences of racism and discrimination; lower symptom and risk factor awareness; language barriers; and culturally insensitive services.^17 18^ This results in later diagnosis, less support, lower likelihood of referral for specialist treatment, and higher morbidity and mortality.^19 20^

This study aims to describe the main reason for attendance to primary care based on routinely collected data entered by the healthcare practitioner; how reason for attendance varies by age, sex, ethnicity and deprivation; and how these factors influence the likelihood of frequent attendance to primary care.

## Methods

This cross-sectional study used anonymised digital general practice records from practices contributing to Lambeth DataNet (LDN). LDN is an electronic database of anonymised primary care records for patients who have permitted to share their records for secondary analysis.

Data were extracted on 29 June 2021, from the period of 1 April 2017–31 March 2020. A year was defined using the financial period from the start of April to the end of March the following calendar year for the purposes of the study. These dates were chosen to exclude the effect of the COVID-19 pandemic on primary care use. At the time of the study, 39 of 40 (95%) practices in Lambeth contributed to LDN; one practice did not contribute as it used an external appointment system.

### Patient and public involvement

Patients were not directly involved in the design of this study. However, this research project was developed alongside LEAP and Lambeth Council, which both identified a need for this research and will be using its findings in their ongoing work within Lambeth Borough.

### Deprivation variable

Deprivation was quantified using an area-level measure, ‘Income Deprivation Affecting Children Index’ (IDACI). IDACI measures in a local area of about 1000–3000 people the proportion of children under the age of 16 years who live in low-income households. It is measured based on the 2019 Lower Layer Super Output Area; or 2011, when 2019 was not listed, linked to the patient via postcode.^21^

As 73% of Lambeth residents live in the two most deprived quintiles in the UK, the IDACI variable is skewed towards the lower quintiles (see table 1).^22^ Therefore, IDACI scores were converted into categorical quintiles specific to Lambeth for the logistic regression. Patients with an unknown IDACI score were removed prior to the logistic regression.

**Table 1:** Summary statistics

Variable	Number (%) of interactions		Number (%) of patients*	
Sex				
Female	45 180 (47.02)		10 611 (47.82)	
Male	50 898 (52.98)		11 578 (52.18)	
Age (years)				
0	17 279 (17.98)		6695 (30.17)	
1	29 645 (30.86)		10 067 (45.37)	
2	19 848 (20.66)		8156 (36.76)	
3	15 682 (16.32)		6866 (30.94)	
4	13 642 (14.18)		6240 (28.12)	
IDACI quintile†				
First	37 761 (39.30)		8731 (37.73)	
Second	33 345 (34.71)		7776 (35.04)	
Third	14 816 (15.42)		3567 (16.08)	
Fourth	8019 (8.35)		1864 (8.40)	
Fifth	2004 (2.09)		574 (2.59)	
Unknown	133 (0.14)		37 (0.17)	
Year				
17–18	34 205 (35.60)		12 552 (56.57)	
18–19	32 374 (33.70)		12 026 (54.20)	
19–20	29 499 (30.70)		11 516 (51.20)	
Ethnicity				
Asian or Asian British (all)	5005 (5.21)		1047 (4.72)	
Indian	920 (0.96)		188 (0.85)	
Pakistani	928 (0.97)		198 (0.89)	
Bangladeshi	1000 (1.04)		162 (0.73)	
Chinese	497 (0.52)		108 (0.49)	
Other	1660 (1.73)		391 (1.76)	
Black or black British (all)	17 299 (18.01)		4062 (18.31)	
African	8530 (8.88)		1981 (8.63)	
Caribbean	4002 (4.17)		963 (4.34)	
Other	4767 (4.96)		1118 (5.04)	
White (all)	29 497 (30.68)		7345 (33.08)	
English, Welsh, Scottish, Northern Irish or British	19 300 (20.09)		4875 (21.97)	
Irish	428 (0.45)		108 (0.49)	
Other	9745 (10.14)		2358 (10.63)	
Mixed or multiple ethnic groups (all)	9620 (10.01)		2254 (10.16)	
White and Black Caribbean	2492 (2.59)		576 (2.60)	
White and Black African	1672 (1.74)		384 (1.73)	
White and Asian	1353 (1.41)		307 (1.38)	
Other	4103 (4.27)		987 (4.45)	
Other ethnic groups (all)	1524 (1.58)		453 (2.05)	
Arab	‡		‡	
Gypsy or Irish Traveller	‡		‡	
Other	1442 (1.50)		434 (1.96)	
Unknown	33 157 (34.51)		7032 (31.69)	
Total	96 078 (100)		22 189 (100)	

* Note sSome of the percentages add to over 100%; this is because the patients returned to the GP for multiple visits in different years and at different ages.

† IDACI quintile – —where 1stfirst quintile represents children living in areas of greatest deprivation and 5thfifth represents those living in the least deprived areas.

‡ Data removed to maintain anonymity.

GPgeneral practitionerIDACIIncome Deprivation Affecting Children Index

### Ethnicity variable

Ethnicity was primarily divided into five categories: Asian or Asian British, black or black British, mixed, other and white, based on the ‘five-plus-one’ ethnic categories used in the 2001 UK national census. However, to allow more detailed understanding of the influence of ethnicity, it was also defined using the 18 ethnicity categories used in the 2011 census when feasible: when an ethnicity category represented over 4% of interactions or were significant at the 5% level in the logistic regression. The two smallest groupings (under 20 children in total) were combined into ‘other’.

### Sex variable

Sex was based on observed sex at birth.

### Age variable

Age is the year of age of the patient at the time of the interaction. Within LDN, only the year of birth is recorded to maintain patient confidentiality. Age was not included as an explanatory variable in the logistic regression due to this imprecision in its calculation and as it does not remain constant across the 3 years. This imprecision may be particularly relevant to this age group due to the rapid rate of change at this age.

### Reason for attendance

Every patient interaction appears as an appointment, with individual codes for different aspects of an appointment. To identify multiple codes relating to one interaction, all codes entered on the same day for a patient were grouped as a single interaction. This means that two appointments on the same day would appear as one.

A coding schema was created to identify reason for attendance using the codes entered within the patient interaction. Therefore, codes relating to administration (for example, when text messages or letters were sent), vaccinations or other routine check-ups were excluded from analysis. Codes were also excluded if they related to procedures, referrals, history taking, examinations, medication, tests and investigations, as while these could give more information, they are too non-specific to determine presenting complaint. Some codes were grouped to avoid overcounting if they were overlapping and redundant—for example, ‘infantile colic’ and ‘abdominal colic’. Codes were also grouped if there were similar complaints referring to separate body parts, such as ‘umbilical pain’, ‘lower abdominal pain’ and ‘abdominal pain’—in this instance, while these could suggest different differentials, they overlap, so were grouped under ‘abdominal pain’. This resulted in a coding schema with 489 secondary-level codes used to identify presenting complaint. Codes related to ‘travel’ were included, as these are appointments requested by the patient to discuss travel including travel vaccines, as opposed to routine appointments.

Multiple complaints were coded in a single interaction. After grouping similar codes, duplicate codes were removed and analysis was conducted on all remaining codes; this allowed more than one complaint to occur within a single interaction. This was because there was no way of identifying the ‘primary’ code/complaint, and analysing the first code entered was considered arbitrary and could miss the existence of multiple complaints. This approach was considered the most effective way to limit overcounting, maximise available data and enable more than one presenting complaint to be identified.

### Analyses

Analyses were conducted using Stata V.14 (StataCorp, College Station, Texas, USA).

The presenting complaint was identified using the codes entered during the patient interaction. The percentage frequency of each code was ranked. The most common complaints representing 50% of interactions were then compared by ethnicity, deprivation, sex, year and age; 10 conditions were compared to enable consistent comparisons despite slight variations in cumulative frequency.

Frequent attendance was defined using the patient as the unit of analysis. The number of interactions linked to a single patient ID was counted. There is no generally accepted definition of how many appointments constitute a frequent attendee; it is typically defined as the top 10–20% of attendances in the population.^23^,^25^ For the purposes of this study, frequent attendees were defined as those within the top 20% in order to maintain power of the study.

To understand frequent attendance, a simple logistic regression was run for each explanatory variable; variables with significant results (at the 5% level) were included in a single adjusted model. SEs were adjusted for clustering by patient.^26^ Explanatory variables used were ethnicity, sex and IDACI. The outcome variable was whether the patient was a frequent attender (in the top 20% of attendances).

## Results

Between 1 April 2017 and 31 March 2020, there were 203 728 patient interactions and 27 923 patients under 5 years old, with 6925 unique codes used (see online supplemental figure 1). Of these, 107 650 interactions did not have a code relating to a presenting complaint (these were mostly administrative or routine examinations) and so were excluded. Once these were removed, there were 121 516 codes within 96 078 interactions for 22 189 patients. While 81.29% of interactions only had one presenting complaint, some interactions had up to 17; 91.05% were unique within the interaction. After duplicate codes within a single interaction were removed, there were 110 637 codes.

### Reason for attendance

Only nine codes made up over 50% of the reason for attendance to primary care for children under 5 years; 29 codes made up 75% of interactions, from 489 secondary-level codes in the study. Upper respiratory tract infection (URTI) was the most common presenting complaint (13.55%), followed by eczema (8.44%) and cough (7.45%) (see table 2). Other frequently occurring conditions included rash (6.32%), fever (3.54%), otitis media (2.92%) and conjunctivitis (2.89%). ‘Rash’ does not include purpura or petechial rashes; it refers to non-specific blanching rashes and urticaria. The code for viral disease (3.8%) also appeared frequently; this non-specific code is often used for fever or flu-like symptoms without an obvious upper respiratory tract involvement, although can sometimes be used for viral rashes with systemic illness.

**Table 2:** Presenting complaints within Lambeth general practices for children under 5 years, ranked by percentage frequency (%) of complaint within the dataset

Rank	Reason for attendance	Percentage frequency of complaint (%)	Cumulative frequency (%)
1	URTI	13.55	13.55
2	Eczema	8.44	21.99
3	Cough	7.45	29.44
4	Rash	6.32	35.76
5	Viral disease	3.80	39.56
6	Fever	3.54	43.10
7	Otitis media	2.92	46.02
8	Conjunctivitis	2.89	48.91
9	LRTI	2.31	51.22
10	Tonsillitis	2.20	53.42
11	Constipation	2.02	55.43
12	Travel	1.96	57.39
13	Diarrhoea and vomiting	1.73	59.12
14	Diarrhoea	1.72	60.84
15	Wheeze	1.55	62.39
16	Vomiting	1.33	63.72
17	Tinea	1.17	64.89
18	Sore throat	1.12	66.00
19	Varicella zoster	1.11	67.11
20	Oral candidiasis	1.05	68.17
21	Reflux	1.02	69.19
22	Hand, foot and mouth disease	0.87	70.06
23	Rhinitis	0.83	70.89
24	Asthma	0.80	71.69
25	Bronchiolitis	0.75	72.44
26	Hernia	0.75	73.18
27	Lymphadenopathy	0.74	73.92
28	Reduced appetite	0.67	74.59
29	Child protection	0.64	75.23
30	Other	24.77	100.00

LRTIlower respiratory tract infectionURTIupper respiratory tract infection

### Sex variable

There was minor variation in the most common conditions between the sexes, with a slightly higher occurrence of conjunctivitis and constipation in female children, and higher rates of tonsillitis in male children (see online supplemental table A).

### Year variable

There was little difference in the most common presenting complaint between each year of study (see online supplemental table B).

### Deprivation variable

When comparing by IDACI quintile, the incidence of eczema was higher for children living in areas of greater deprivation (see table 3).

**Table 3:** Most common 10 condition codes ranked by percentage frequency of complaint within the dataset, separated by IDACI quintile of patient (first quintile represents children living in areas of greatest deprivation and fifth represents those living in the least deprived area)

Rank	Most common 10 condition codes separated by IDACI quintile of patient				
	First quintile (%)	Second quintile (%)	Third quintile (%)	Fourth quintile (%)	Fifth quintile (%)
1	URTI (14.02)	URTI (13.44)	URTI (13.43)	URTI (12.2)	URTI (12.45)
2	Eczema (9.01)	Eczema (8.52)	Cough (7.56)	Cough (8.37)	Cough (7.23)
3	Cough (7.15)	Cough (7.54)	Eczema (7.5)	Eczema (7.56)	Eczema (6.75)
4	Rash (6.13)	Rash (6.48)	Rash (6.24)	Rash (6.67)	Rash (6.49)
5	Viral disease (3.73)	Viral disease (3.72)	Viral disease (3.8)	Viral disease (4.03)	Viral disease (5.57)
6	Fever (3.66)	Fever (3.49)	Otitis media (3.33)	Otitis media (3.64)	Otitis media (3.92)
7	Conjunctivitis (2.66)	Conjunctivitis (2.94)	Fever (3.31)	Fever (3.59)	Conjunctivitis (3.87)
8	Otitis media (2.62)	Otitis media (2.85)	Conjunctivitis (2.93)	Conjunctivitis (3.49)	Fever (3.53)
9	Travel (2.23)	Tonsillitis (2.27)	LRTI (2.7)	LRTI (3.05)	Constipation (2.44)
10	LRTI (2.19)	LRTI (2.14)	Tonsillitis (2.64)	Tonsillitis (2.13)	Tonsillitis (2.31)

IDACIIncome Deprivation Affecting Children IndexLRTIlower respiratory tract infectionURTIupper respiratory tract infection

### Ethnicity variable

When comparing presenting complaint by ethnicity, URTI remained the most common complaint, except for children of Caribbean ethnicity, where eczema was the most common complaint. Eczema remained the second most common complaint for all other ethnicities except white British and other, where it was the third and fourth most common, respectively (table 4). Tinea, a fungal skin infection, was more common for African, Caribbean and other white ethnicities. Constipation was also a more common reason for patient interaction for Asian, African, Caribbean and other white ethnicities (see online supplemental table C).

**Table 4:** Most common 10 condition codes ranked by percentage frequency of complaint within the dataset, separated by 5+1 categories of ethnicity

Most common 10 condition codes, separated by 5+1 categories of ethnicity						
Rank	White (%)	Asian (%)	Black (%)	Mixed (%)	Other (%)	Unknown (%)
1	URTI (13.45)	URTI (14.4)	URTI (12.82)	URTI (13.74)	URTI (12.83)	URTI (13.86)
2	Cough (7.88)	Eczema (10.73)	Eczema (11.22)	Eczema (8.55)	Cough (7.49)	Eczema (8.46)
3	Rash (6.79)	Cough (6.22)	Cough (6.79)	Cough (7.21)	Eczema (7.21)	Cough (7.68)
4	Eczema (6.39)	Rash (4.61)	Rash (5.85)	Rash (7.01)	Rash (6.13)	Rash (6.23)
5	Viral disease (4.11)	Fever (4.56)	Viral disease (3.29)	Viral disease (3.78)	Fever (3.97)	Viral disease (3.79)
6	Fever (3.99)	Travel (3.89)	Fever (3.11)	Fever (3.2)	Viral disease (3.86)	Fever (3.28)
7	Otitis media (3.72)	Viral disease (3.89)	Travel (3)	Otitis media (2.86)	Diarrhoea and vomiting (2.89)	Conjunctivitis (2.84)
8	Conjunctivitis (3.53)	Tonsillitis (2.81)	Constipation (2.38)	Conjunctivitis (2.69)	Conjunctivitis (2.67)	Otitis media (2.72)
9	Tonsillitis (2.45)	Otitis media (2.72)	Conjunctivitis (2.34)	LRTI (2.44)	Diarrhoea (2.67)	Tonsillitis (2.33)
10	LRTI (2.41)	Constipation (2.37)	LRTI (2.19)	Tonsillitis (2)	Otitis media (2.67)	LRTI (2.31)

LRTIlower respiratory tract infectionURTIupper respiratory tract infection

### Age variable

Reflux, hernias and oral candidiasis are much more common complaints for children under 1 year. Complaints from children 1–4 years old remain largely similar in comparison, with some exceptions (see online supplemental table D).

### Frequent attendance

The median number of interactions was 3 per patient across the 3 years (IQR 5). The highest number of interactions for a single patient across 3 years was 68; this could be for new complaints or ongoing issues.

### Sex variable

Male sex was not significantly associated with frequent attendance within the univariate regression (OR 1.02 (0.95 to 1.09)) (see online supplemental table E). Therefore, sex was not included in the multivariate regression.

### Deprivation variable

Children living in the most deprived areas had 1.27 (1.14 to 1.41) times increased odds of being a frequent attender (seven or more interactions in 3 years) compared with those living in the least deprived areas, after adjusting for the effects of ethnicity (see table 5).

**Table 5:** Two multivariate logistic regression models for frequent attendance, using ethnicity and IDACI quintile as explanatory variables

Variable	Adjusted OR (95% CI)	P value
Model 1: adjusted for ethnicity and deprivation, using the 5+1 categories of ethnicity and Lambeth-specific IDACI quintile*		
IDACI quintile*		
First	**1.27 (1.14 to1.41**)	**<0.001**
Second	**1.16 (1.05 to1.29**)	**0.005**
Third	**1.27 (1.15 to1.41**)	**<0.001**
Fourth	0.97 (0.88 to 1.08)	0.610
Fifth†	1	–
Ethnicity		
White†	1	–
Asian	**1.38 (1.18 to1.61**)	**<0.001**
Black	1.04 (0.94 to 1.15)	0.469
Mixed	1.08 (0.96 to 1.22)	0.207
Other	**0.59 (0.44 to0.79**)	**<0.001**
Unknown	**1.33 (1.23 to1.44**)	**<0.001**
Model 2: adjusted for ethnicity and deprivation, using the 18 categories of ethnicity and Lambeth-specific IDACI quintile		
IDACI quintile*		
First	**1.25 (1.12 to1.39**)	**<0.001**
Second	**1.14 (1.03 to1.27**)	**0.012**
Third	**1.26 (1.14 to1.40**)	**<0.001**
Fourth	0.97 (0.87 to 1.07)	0.508
Fifth†	1	–
Ethnicity		
White British†	1	–
Other white	**1.18 (1.04 to1.34**)	**0.009**
Irish	1.24 (0.77 to 2.00)	0.368
Gypsy or Irish Traveller	1.67 (0.17 to 16.63)	0.661
Bangladeshi	**2.70 (1.95 to3.74**)	**<0.001**
Chinese	1.20 (0.75 to 1.92)	0.453
Indian	**1.47 (1.04 to2.08**)	**0.030**
Pakistani	1.35 (0.96 to 1.90)	0.089
Other Asian	1.18 (0.91 to 1.53)	0.219
African	1.14 (0.99 to 1.30)	0.060
Caribbean	0.99 (0.83 to 1.19)	0.914
Other black	1.15 (0.98 to 1.36)	0.095
White and Asian	1.20 (0.90 to 1.60)	0.220
White and black African	1.21 (0.94 to 1.57)	0.144
White and black Caribbean	1.18 (0.95 to 1.47)	0.137
Other mixed	1.09 (0.92 to 1.30)	0.325
Other	**0.60 (0.44 to0.81**)	**0.001**
Arab	1.58 (0.50 to 5.00)	0.434
Unknown	**1.41 (1.29 to1.55**)	**<0.001**

Results significant at the 5% level (p<0.05) in bold.

* Where first quintile represents children living in areas of greatest deprivation and fifth represents those living in the least deprived area.

† Reference group.

IDACIIncome Deprivation Affecting Children Index

### Ethnicity variable

In the univariate regression model, children of Asian (OR 1.39 (1.19 to 1.63)), black (OR 1.10 (1.00 to 1.22)) and unknown (OR 1.36 (1.25 to 1.48)) ethnicities were more likely to be frequent attenders to primary care (seven or more interactions in 3 years) relative to children of white ethnicity. Relative to children of white ethnicity, children of ‘other’ ethnicity had 0.59 (0.44 to 0.79) times the odds of frequent attendance to primary care during the study.

After adjusting for deprivation, children of Asian ethnicity had 1.38 times (1.18 to 1.61) increased odds of being a frequent attendee relative to children of white ethnicity. Children of black ethnicity were no longer significantly more likely to be frequent attenders (OR 1.04 (0.94 to 1.15)).

When using a more detailed breakdown for ethnicity, children of Bangladeshi (OR 2.70 (1.95 to 3.74)), Indian (OR 1.47 (1.04– to 2.08)) and other white (OR 1.18 (1.04 to 1.34)) ethnicities all had increased odds of being frequent attenders to primary care compared with white British children, after adjusting for deprivation, significant at the 5% level. Children of ‘unknown’ ethnicity remained significantly likely to be a frequent attendee (OR 1.41 (1.29 to 1.55)); however, it is difficult to draw inferences from this as we do not know which children were included within this group, as with children of ‘other’ ethnicity. Children of ‘other’ ethnicity remained less likely to be frequent attenders of primary care (OR 0.60 (0.44 to 0.81)).

## Discussion

### Main findings

#### Reason for attendance to primary care

A small number of conditions create a disproportionate demand on primary practice: 9 conditions make up over half of interactions to primary care for children under 5 years old and 29 conditions form over three-quarters. Most are related to acute viral infections, many of which are self-limiting (resolve without medical intervention) and could potentially be managed within the community, via self-management and community allied health professionals, such as pharmacists and health visitors.

#### Social determinants influencing attendance to primary care

The disease profile in the first year of life is substantially different to the following 4 years; reflux is far more common, as is oral candidiasis and hernia. Eczema is a far more common reason for attendance in children of non-white ethnicity, in particular black Caribbean children and children living in areas of greater deprivation. Tinea was also a more frequent complaint among African, Caribbean and other white ethnicity in this study. However, the nine conditions identified earlier remain dominant.

#### Factors influencing frequent attendance

The effect of ethnicity on frequent attendance in this study is complex; these analyses suggest non-white British ethnicity increases the likelihood of being a frequent attendee, but this effect is not consistent for all ethnicities. It also highlights the importance of using a more detailed ethnicity breakdown, for example, children of Bangladeshi ethnicity are more likely than the other Asian ethnicities to be a frequent attender. Age has not been adjusted for in the regression, but previous research suggests younger age is associated with frequent attendance; black and Asian ethnicities have a slightly older demographic than white ethnicities, so age may be acting as a negative confounder obscuring the observed relationship.^7^

### Interpretation

The management pathway for URTI, acute otitis media, acute cough symptoms, tonsillitis and many viral rashes include self-management or ‘watch and wait’ approaches.^27^,^30^ There are, of course, potential complications and ‘red flag’ symptoms indicating serious disease; however, it is likely the vast majority would not require medical attention.^31^ This may indicate that caregivers require greater support to manage these conditions at home and that there is an opportunity to build capacity within community resources to provide caregivers support.

Greater knowledge is required to understand the psychological motivators in attendance, including factors such as parental anxiety. Reflux, also known as posseting, is reported in 67% of infants under 4 months and does not usually require investigation.^32^ Despite this, previous research indicates a high level of parental anxiety associated with reflux, with 24% raising concerns at well-baby visits and was identified as a common complaint in this study.^33^ Hernias were also identified as a common presentation in children under 1 year: the majority of these will be congenital umbilical hernias and will resolve given time.^34^ Therefore, despite a different disease profile to the other age groups, again the majority of primary care presentations for children under 1 year could be reduced by providing alternative means of advice. Some alternative support for some of these conditions already exists, such as health visitors or local breastfeeding support groups. However, these may not be widely known about, may not provide the same reassurance to parents, and provision of these may not be equitable among all communities and may require greater signposting.

These minor conditions may have a more significant impact on these children’s development than is immediately apparent: for example, infant oral candidiasis is associated with maternal nipple candidiasis, which can result in severe maternal breast pain and premature weaning.^35^ These infections are more common with improper cleaning of dummies; they can be managed by pharmacists after 4 months of age.^36 37^ Simple health promotion campaigns may provide sufficient knowledge to caregivers to prevent this from occurring, with the co-benefit of reducing premature weaning. Alternatively, this may be related to a lack of resources within the home environment to keep these items clean; this is where a larger study could provide greater insights into the motivators behind these findings, as it could allow breakdowns by age and deprivation for example.

The conditions identified in this study could provide a focus point for GP training, to improve the management of these conditions and better identification of those who may need secondary care. Currently, the limited formal training that some GPs receive occurs via hospital placements within paediatric departments; as this study has identified, the majority of paediatric complaints within primary care are minor, self-limiting conditions and so are unlikely to be managed within secondary care.^11^ Paediatric GP training could include emphasising the primary care physician’s role as an ‘educator’ for caregivers, to improve self-management and reduce repeat attendances for minor conditions.

Children of non-white British ethnicities may be more likely to be frequent attendees for several reasons, including cultural differences in healthcare use; increased likelihood of living in an area of greater deprivation; the effect of migration reducing support network size; perceived accessibility of community services or because of a higher burden of disease.^16 38 39^ There is also an established evidence base to suggest that minoritised ethnicities are less likely to be referred to specialist treatment or receive medication in primary care than their white counterparts.^40^ Therefore, children of non-white British ethnicities may have to attend multiple times in order to receive treatment.

After 5 years old, 60–80% of children will not experience eczema symptoms; however, one in every three children with eczema will develop asthma or allergic rhinitis, meaning eczema is associated with a large burden of illness.^41^ Previous studies have found eczema is more prevalent among black Caribbean children in London compared with white children, which is consistent with the findings of this study.^42^

Previous studies have also suggested that eczema is more common among children of higher socioeconomic status; the greater number of attendances related to eczema among children living in areas of greater deprivation in this study may be confounded by ethnicity.^42^,^44^ Nonetheless, people experiencing greater deprivation and of non-white ethnicity also experience lower rates of specialist referral and less potent treatment prescribed—the potentially poorer quality of care received may result in a higher number of appointments.^14^ There may also be differences in the severity of eczema, resulting in a higher number of attendances. The intersectionality between different sociodemographic factors, such as ethnicity and deprivation, was not accounted for in the analysis of presenting complaint, due to size limitations of the dataset.

An increased incidence of tinea infections is also associated with crowded living conditions and greater deprivation, particularly within urban populations.^45^ Tinea capitis is more common among black children, as found in study, the reason for which is unknown; although black African and Caribbean children are also more likely to live in areas of higher deprivation and overcrowded housing.^39^ There is an abundance of evidence also linking eczema to poor-quality housing, which could suggest the impact of housing on healthcare use in this population.^46^ This perhaps highlights the links between these social determinants and a limitation in reducing such a significant factor, such as deprivation, to a single measure.

### Limitations

This study used a large dataset to analyse reason for attendance. Lambeth’s large, diverse population enabled detailed analysis by ethnicity, which has previously been lacking from the literature. However, as this study was conducted within a relatively small geographical area, this may limit the generalisability of the findings. There may be characteristics that are specific to Lambeth, including that it is more ethnically diverse than many areas of the UK.^47^ This may influence the effects of ethnicity and racism on health and healthcare use as detected by this study, which reduce the generalisability of these findings to the rest of the UK.

This study used the ethnic groups from the 2001 and 2011 census, as those were the groups recorded by the patient and this also enables comparison with other (past/future) data sources. There have been criticisms of these ethnic groupings; however, in terms of how they homogenise diverse experiences of culture and racism are based on largely arbitrary distinctions.^48^ Further research is required to understand the diverse experiences and drivers that may or may not go along ethnic group lines in order to fully understand these influences and the limitations of research using these categories.

The most significant limitation of this study is the inability to distinguish differences between the burden of disease and differences in health services use. Frequent attendance could be related to increased health anxiety in some communities, reduced support or a higher burden of disease. It could also be linked to greater severity of disease or poorer treatment outcomes within the appointment. Similarities in reason for appointment between groups may be due to similar disease profiles or may be due to cultural norms dictating when to use primary care. Potentially, qualitative analysis of caregivers’ motivations to attend may provide more insight into health behaviours, particularly if stratified by broader characteristics to understand trends in behaviour. The influence of health cultures on health behaviours and outcomes, and therefore the ability to mitigate or heighten inequalities, should not be underestimated. There is a need for culturally competent services to work with communities to understand and deliver health services, providing an opportunity to reconcile divergent health perspectives between service provider and user. While it is not within the scope of this paper to explore this, there are multiple models that have attempted to bridge this divide, such as the ‘Explanatory Models Approach’.^49^

This study removed ‘routine’ appointments, in order to isolate and focus on patient-requested appointments or health concerns. This was an attempt to conceptualise what patients were requesting from their primary care services. However, this prevented the study from comparing different uptake of primary interventions, such as routine national vaccinations. This may have explained some of the variation in attendance between different groups, for example, if lower uptake of vaccinations led to an increase in presentation for vaccine-preventable conditions.

### Future research

Greater knowledge of motivators of attendance, the outcome of appointments and prevalence of disease for this age group is required to understand why certain groups attend primary care more frequently than others. Future health promotion attempts require greater cultural sensitivity to adequately engage with these communities, with a greater focus on structural determinants influencing attendance for these groups. Additionally, a greater understanding of the efficacy of current health promotion attempts is warranted. This includes the Healthy Child Programme, part of which involves signposting families to community services and resources.^50^ For example, families may not be aware that they can continue to contact their Health Visitor until their child is 5 years old for many of the conditions identified in this study, outside of mandatory visits. Furthermore, the ‘Family Hub’ Initiative has recently been developed to provide support for families with children aged 0–19 years along certain key public health deliverables, such as infant feeding, with a focus on health inequality; it will be interesting to observe how this programme influences the outcomes identified in this study.^51^

There may be substantial differences in the disease burden and profile in rural areas compared with inner-city London; the effect of structural inequality may be different in urban and rural locations, due to different resources, accessibility and community networks. Furthermore, there is significant variation in markers of child health across the country, including infant mortality, suggesting that this study should be repeated in different geographical locations to provide further insight into this phenomenon.^52^

Further research is required to build on the findings of this study to fully quantify and conceptualise its impact. This demonstrates some of the potential uses of primary care databases, such as LDN, to study, monitor and manage primary care services. The impact of the COVID-19 pandemic will have an unknown effect on child usage of primary care, and how this will continue to affect use in the future. Future studies combining caregiver and child data may further understanding of how health services are used and what influences their use, for example, whether frequent attendance by caregivers is associated with frequent attendance for children. It may also identify correlations between certain conditions for an individual and an increase or decrease in net attendance for a family unit: for example, whether a diagnosis of generalised anxiety disorder in a caregiver increases attendance for the whole family unit.

## Conclusion

A small number of acute, self-limiting conditions form a large burden on primary care, the majority of which could be managed elsewhere. There are minor variations in the reason for attendance by ethnicity and age; however, the social determinants of health did not affect the reason for attendance as much as suspected. The social determinants of health did affect the frequency of attendance: factors influencing the likelihood of frequent attendance include living in an area of greater deprivation, and Bangladeshi, Indian or other white ethnicity.

To promote sustainable use of health services, local authorities may need to consider how community care is delivered to this age group. This includes alternative means of treatment for minor ailments, as well as ensuring health promotion messages are co-developed with these communities.

## supplementary material

10.1136/bmjopen-2023-082253 online supplemental file 1

## Data Availability

No data are available.
