# Supplementary material for: Why do children under 5 years go to the GP in Lambeth: a cross-sectional study
Source: BMJ Open. 2024 May 23;14(5):e082253. doi: 10.1136/bmjopen-2023-082253 (PMC11328670; doi:10.1136/bmjopen-2023-082253)
Supplement: online supplemental file 1 [file bmjopen-14-5-s001.pdf]

Supplementary Table A: **MOST COMMON TEN CONDITION CODES, SEPARATED BY SEX**

**RANK**	**MOST COMMON TEN CONDITION CODES, SEPARATED BY SEX**			
	Female (n=)	**Percentage Frequency of code (%)**	Male (n=)	**Percentage Frequency of code (%)**
**1**	URTI	13.67	URTI	13.44
**2**	Eczema	8.93	Eczema	8.01
**3**	Cough	7.56	Cough	7.36
**4**	Rash	6.58	Rash	6.09
**5**	Viral disease	3.86	Viral disease	3.75
**6**	Pyrexia	3.55	Pyrexia	3.53
**7**	Conjunctivitis	2.91	Otitis media	3.05
**8**	Otitis media	2.77	Conjunctivitis	2.88
**9**	Constipation	2.31	LRTI	2.34
**10**	LRTI	2.28	Tonsilitis	2.33

Supplementary Table B: **MOST COMMON TEN CONDITION CODES, SEPARATED BY TIME PERIOD THAT APPOINTMENT OCCURRED WITHIN**

**RANK**	**MOST COMMON TEN CONDITION CODES, SEPARATED BY TIME PERIOD THAT APPOINTMENT OCCURRED WITHIN**							
	**2017-18**	**Percentage Frequency of code (%)**	**2018-19**	**Percentage Frequency of code (%)**	**2019-20**	**Percentage Frequency of code (%)**	**2017-2020**	**Percentage Frequency of code (%)**
**1**	URTI	13.42	URTI	13.74	URTI	13.48	URTI	13.55
**2**	Eczema	8.99	Eczema	8.42	Eczema	7.83	Eczema	8.44
**3**	Cough	7.48	Cough	7.34	Cough	7.54	Cough	7.45
**4**	Rash	6.61	Rash	6.42	Rash	5.88	Rash	6.32
**5**	Viral disease	3.77	Viral disease	3.84	Viral disease	3.79	Viral disease	3.8
**6**	Pyrexia	3.67	Pyrexia	3.42	Pyrexia	3.52	Pyrexia	3.54
**7**	Conjuncti vitis	2.93	Otitis media	3.06	Otitis media	3.01	Otitis media	2.92
**8**	Otitis media	2.7	Conjunctivitis	2.92	Conjunctivitis	2.81	Conjunctivitis	2.89
**9**	Tonsilitis	2.14	LRTI	2.48	LRTI	2.59	LRTI	2.31
**10**	Constipat ion	2.01	Tonsilitis	2.09	Tonsilitis	2.4	Tonsilitis	2.2

Supplementary Table C: **MOST COMMON TEN CONDITION CODES RANKED BY PERCENTAGE FREQUENCY OF COMPLAINT WITHIN THE DATASET, SEPARATED BY 18 CATEGORIES OF ETHNICITY (WHEN CATEGORY REPRESENTS >4% OF THE SAMPLE)**

	**MOST COMMON TEN CONDITION CODES, SEPARATED BY THE 18 CATEGORIES OF ETHNICITY (WHEN CATEGORY REPRESENTS >4% OF THE SAMPLE)**											
**RANK**	**African**	**Percent-age (%)**	**Caribbean**	**Percent-age (%)**	**Other Black**	**Percent-age (%)**	**White British**	**Percent-age (%)**	**Other White**	**Percent-age (%)**	**Other Mixed**	**Percent-age (%)**
**1**	URTI	13.49	Eczema	14.11	URTI	12.38	URTI	12.66	URTI	12.38	URTI	13.96
**2**	Eczema	9.62	URTI	11.9	Eczema	11.69	Cough	7.98	Eczema	11.69	Eczema	8.13
**3**	Cough	6.61	Rash	7.31	Cough	7.35	Rash	7.02	Cough	7.35	Cough	7.98
**4**	Rash	5.33	Cough	6.52	Rash	5.55	Eczema	6.66	Rash	5.55	Rash	7.03
**5**	Travel	4.12	Viral disease	3.36	Viral disease	3.42	Viral disease	4.00	Viral disease	3.42	Viral disease	4.09
**6**	Pyrexia	3.45	Conjuncti-vitis	2.88	Pyrexia	3.04	Conjuncti-vitis	3.78	Pyrexia	3.04	Otitis media	3.1
**7**	Viral disease	3.18	Constipa-tion	2.6	LRTI	2.8	Otitis media	3.78	LRTI	2.8	Conjuncti-vitis	2.95
**8**	Constipa-tion	2.27	Pyrexia	2.47	Travel	2.68	Pyrexia	3.69	Travel	2.68	Pyrexia	2.93
**9**	Conjuncti-vitis	2.23	Tinea	2.04	Constipa-tion	2.38	LRTI	2.4	Constipa-tion	2.38	Tonsilitis	2.3
**10**	Tinea	2.03	LRTI	1.98	Tinea	2.37	Tonsilitis	2.21	Tinea	2.37	LRTI	2.17

Supplementary Table D: **MOST COMMON TEN CONDITION CODES RANKED BY PERCENTAGE FREQUENCY OF COMPLAINT WITHIN THE DATASET, SEPARATED BY AGE OF PATIENT (YEARS) AT TIME OF PATIENT INTERACTION**

	MOST COMMON TEN CONDITION CODES RANKED BY PERCENTAGE FREQUENCY OF COMPLAINT WITHIN THE DATASET, SEPARATED BY AGE OF PATIENT (YEARS) AT TIME OF PATIENT INTERACTION									
**RANK**	**0 years**		**1 year**		**2 years**		**3 years**		**4 years**	
**1**	URTI	11.72	URTI	15.75	URTI	14.59	URTI	12.52	URTI	10.75
**2**	Eczema	10.28	Eczema	9.66	Eczema	8.08	Cough	7.97	Cough	7.53
**3**	Rash	5.95	Cough	7.86	Cough	8.06	Eczema	6.82	Eczema	5.96
**4**	Cough	5.47	Rash	7.44	Rash	6.5	Rash	5.52	Rash	5.06
**5**	Reflux	3.99	Viral disease	4.46	Viral disease	4.09	Pyrexia	4.13	Tonsilitis	3.63
**6**	Oral candidiasis	3.71	Pyrexia	3.99	Pyrexia	4.03	Viral disease	3.81	Pyrexia	3.52
**7**	Conjunctivitis	3.43	Conjunctivitis	3.46	Otitis media	3.87	Otitis media	3.42	Otitis media	3.23
**8**	Hernia	2.83	Otitis media	3.1	Conjunctivitis	3.1	Tonsilitis	3.32	Viral disease	3.22
**9**	Viral disease	2.79	LRTI	2.57	LRTI	2.88	Travel	2.59	Asthma	2.97
**10**	Constipation	2.78	Diarrhoea	2.42	Tonsilitis	2.75	LRTI	2.59	Travel	2.77

Supplementary Table E: Univariate logistic regression of frequent attendance. *Reference group. Results significant at the 5% level (p<0.05) in bold.

**Variable**		**Univariate OR (95% CI)**	***P-value***
Sex	Female*	1	-
	Male	1.02 (0.95-1.09)	*0.605*
(Lambeth-specific)	First	1.28 (1.15-1.42)	***<0.001***
IDACI quintile	Second	1.16 (1.05-1.28)	**0.005**
	Third	1.28 (1.15-1.41)	**<0.001**
	Fourth	0.98 (0.88-1.09)	0.692
	Fifth*	1	-
Ethnicity	White*	1	-
	Asian	1.39 (1.19-1.63)	***<0.001***
	Black	1.10 (1.00-1.22)	***0.048***
	Mixed	1.11 (0.98-1.25)	*0.091*
	Other	0.61 (0.45-0.81)	***0.001***
	Unknown	1.36 (1.25-1.48)	***<0.001***
