# Supplementary material for: Why do children under 5 years go to the GP in Lambeth: a cross-sectional study
Source: BMJ Open. 2024 May 23;14(5):e082253. doi: 10.1136/bmjopen-2023-082253 (PMC11328670; doi:10.1136/bmjopen-2023-082253)
Supplement: Supplementary file 2 [file bmjopen-2023-082253.reviewer_comments.pdf]

PEER REVIEW HISTORY

BMJ Open publishes all reviews undertaken for accepted manuscripts. Reviewers are asked to complete a checklist review form (**http://bmjopen.bmj.com/site/about/resources/checklist.pdf**) and are provided with free text boxes to elaborate on their assessment. These free text comments are reproduced below.

ARTICLE DETAILS

**TITLE (PROVISIONAL)**	Why do children under 5 years go to the GP in Lambeth: a cross-sectional study
**AUTHORS**	Craven, Eleanor; Luck, Gemma; Whitney, David; Dodhia, Hiten; Foster, Shaneka; Stanke, Carla; Seed, Paul; Crompton, James; Brown, Kerry Ann

VERSION 1 – REVIEW

**REVIEWER**	Vance, Alasdair<br>University of Melbourne, MDHS
**REVIEW RETURNED**	11-Dec-2023

**GENERAL COMMENTS**	This is a very interesting and well written paper. I think it could be improved if the authors were to provide additional detail on [1] the different cultural belief systems and practices about health and wellbeing across the ethnicities studied as part of the future directions section and [2] the possible evolution of health care services to be more culturally appropriate, safe and engaging for the different ethnicities studied.<br><br>These cultural factors as Tseng (2006) outlines in Transcultural Psychiatry modify the presentation, management, natural history and outcome of mental disorder as well as other health conditions.

**REVIEWER**	Delacroix, Emerson<br>University of Michigan, School of Public Health
**REVIEW RETURNED**	27-Dec-2023

**GENERAL COMMENTS**	General Comments: Well written and well organized paper. The subject of visit reason and inequities is valuable. I appreciate the use of IDACI to discern differences of attendance reasons. I appreciate the ideas of managing acute viral infections, and minor conditions, outside the primary care setting.<br><br>I do not see Paul T Seed listed in Author contributions.<br><br>I would like to know why vaccination visits were not included in analysis, but vaccination for travel was included in the travel category. I'm curious if IDACI, age, or race and ethnicity impacted attendance of routine-care and vaccination visits. Given that children living in more deprived areas has more frequent attendance, I'd expect routine-care visits to be missed in lieu of an anticipated sick-visit.<br><br>Some picky edits:

	- Abstract and Methods: study start date is listed day then month. In the Results study start date is listed as month then day.<br>- Reason for attendance section: an oxford comma is missing after eczema (8.44%).<br>- Social Determinants influencing attendance to primary care: an oxford comma is missing after candidiasis.<br><br>One suggestion to discern parental anxiety vs. burden of disease could be the inclusion of the Generalized Anxiety Disorder 7 (GAD-7) or similar assessment for caregivers at visits.

VERSION 1 – AUTHOR RESPONSE

**Reviewer 1**		
This is a very interesting and well written paper. 2. I think it could be improved if the authors were to provide additional detail on [1] the different cultural belief systems and practices about health and wellbeing across the ethnicities studied as part of the future directions section and [2] the possible evolution of health care services to be more culturally appropriate, safe and engaging for the different ethnicities studied. These cultural factors as Tseng (2006) outlines in Transcultural Psychiatry modify the presentation, management, natural history and outcome of mental disorder as well as other health conditions.	2.These are valid and valuable points. We recognise the limited space to elaborate on the different health cultures of these communities and adequately do justice to this topic. Nevertheless, a paragraph has been included to highlight the importance of this area for future research and policy/practice discussions.	Limitations paragraph 2
**Comments**	**Response**	**Section ref.**
**Reviewer 2**		
General Comments: Well written and well organized paper. The subject of visit reason and inequities is valuable. I appreciate the use of IDACI to discern differences of attendance reasons. I appreciate the ideas of managing acute viral infections, and minor conditions, outside the primary care setting. 3. I do not see Paul T Seed listed in Author contributions.	3. Apologies for the oversight. Paul T Seed's contribution has been added.<br><br>4. & 5. We apologise for the confusion. We believe these two points are linked and have considered in tandem. Vaccination visits were not included; these were considered routine	Authors' contributions<br><br>Limitations paragraph 3

4. I would like to know why vaccination visits were not included in analysis, but vaccination for travel was included in the travel category.<br>5. I’m curious if IDACI, age, or race and ethnicity impacted attendance of routine-care and vaccination visits. Given that children living in more deprived areas has more frequent attendance, I’d expect routine-care visits to be missed in lieu of an anticipated sick-visit.<br><br>Some picky edits:<br>6. Abstract and Methods: study start date is listed day then month. In the Results study start date is listed as month then day.<br><br>7. Reason for attendance section: an oxford comma is missing after eczema (8.44%).<br><br>8. Social Determinants influencing attendance to primary care: an oxford comma is missing after candidiasis.<br><br>9. One suggestion to discern parental anxiety vs. burden of disease could be the inclusion of the Generalized Anxiety Disorder 7 (GAD-7) or similar assessment for caregivers at visits.	appointments. Travel vaccinations were not considered routine appointments, thus included separately. The reasoning for excluding routine appointments and limitations associated with this decision has been clarified in the limitations section as per below.<br><br>“This study removed ‘routine’ appointments, in order to isolate and focus on patient-requested appointments or health concerns. This was an attempt to conceptualise what patients were requesting from their primary care services. However, this prevented the study from comparing different uptake of primary interventions, such as routine national vaccinations. This may have explained some of the variation in attendance between different groups, for example if lower uptake of vaccinations led to an increase in presentation for vaccine-preventable conditions.”<br><br>In addition, for further clarification on the data set presented, we have<br>i) added a footnote to table 1, so it is clear why percentages can be >100% [as patients returned to the GP for multiple visits in different years and at different ages], and<br>ii) clarified the text, see below, referring to IDACI quintiles, so it is clear when and why data represent Lambeth specific quintiles.<br><br>“As 73% of Lambeth residents live in the two most deprived quintiles in the UK, the IDACI variable is skewed towards the lower quintiles (see table 1). Therefore, IDACI scores were converted into categorical quintiles specific to Lambeth for the logistic regression. Patients with an unknown IDACI score were removed prior to the logistic regression.”<br><br>6. Completed in line with comments<br><br>7. Completed in line with comments<br><br>8. Completed in line with comments<br><br>9. Thank you for this insight. This has been added to the future directions section.	Table 1<br><br>Methods, deprivation variable heading<br><br>Results paragraph 1<br><br>Results, reason for attendance<br><br>Discussion paragraph 2<br><br>Future directions paragraph 3

